# Geriatric Medicine in an Aging Society: Up for a Challenge?

**DOI:** 10.3389/fmed.2014.00010

**Published:** 2014-04-29

**Authors:** Arduino A. Mangoni

**Affiliations:** ^1^Department of Clinical Pharmacology, School of Medicine, Flinders Medical Centre, Flinders University, Adelaide, SA, Australia

**Keywords:** aging, geriatric medicine, healthcare systems, independence, workforce

A progressive decrease in fertility, together with lengthening life expectancy, has driven a reshaping in the age distribution of the world population (1). The main consequence of this reshaping is a progressive increase in both the number and the relative proportion of older people in our society. Current fertility rates in virtually all developed countries are below replacement levels, reflecting the significant societal, cultural, and lifestyle changes over the last century. A faster decline in fertility is occurring in developing countries. The latter is anticipated to reduce global geographical differences in fertility rates. The increase in life expectancy is related to a number of factors such as improved socioeconomic conditions and access to health services, early detection of disease conditions, and improved short- and long-term outcomes after an illness. Mean global life expectancy has increased from 46.5 years in 1950–1955 to 66.0 years in 2000–2005. This increase has been more prominent in less developed countries although significant variation exists in these regions (2). Notably, the relative gain in life expectancy is also projected to increase at older age, i.e., by 27% at age 80, 19% at age 60, and only 9% at birth over the next 50 years. Therefore, specific subgroups within the older population, e.g., >80 years, are growing particularly fast.

Geriatric Medicine, as a clinical and academic specialty in its own right, has significantly expanded over the last 20 years, particularly in the UK, a number of Western European countries, North America, Australia, and New Zealand. A recent census in the UK showed that Geriatric Medicine consultants represent the largest specialty group within the National Health Service (*n* = 1,252; 10.2% of the total workforce) (3). Moreover, the number of advanced trainees in Geriatric Medicine has increased from 433 in 2003 to 630 in 2013 (+45%). Professional national and international bodies, e.g., the British and American Geriatrics Societies and the European Union Geriatric Medicine Society, have played a significant role in promoting the importance of a focused geriatric assessment and a multidisciplinary patient-centered approach, key pillars of old age care. Moreover, they have been instrumental in developing and refining national curricula and postgraduate training competencies as well as continuing professional development opportunities. A considerable work to raise awareness and interest in Geriatric Medicine has also been conducted in medical schools. There is some evidence that targeted teaching and learning activities might increase the likelihood of undergraduate students to consider a career in Geriatric Medicine (4). At first glance, the expansion of the specialty seems commensurate to the progressive increase in the number of older adults worldwide (2). However, dedicated care of older adults has barely developed in many other countries. This picture is unlikely to change substantially within the next 10 years.

Geriatricians often subspecialize in areas of interest and/or local needs, e.g., falls, dementia, and incontinence services. Yet, they maintain solid collaborative links with other specialties, e.g., orthopedics, neurology, and psychiatry, and perform their duties both in hospital and in the community. Although this multitasking approach is laudable, there are increasing concerns about the capacity of the specialty to consistently deliver excellent care in so many different domains. A key factor is represented by the increasing short- and long-term needs of older people. Such needs go far beyond the pharmacological and non-pharmacological management of acute and chronic disease conditions and depend, at least in part, on a number of factors directly associated with the aging process itself. First, there is a progressive reduction in the homeostatic capacity of different organs and systems (5). This might be due to a “physiologic” reduction in organ function, e.g., glomerular filtration rate, as well as co-morbid conditions and/or use of medications affecting organ function *per se*. As a result, the capacity of older adults to recover after an illness can be significantly affected. This might lead to a prolonged hospital stay, a rehabilitation period, or the need for ongoing medical and/or social support in the community. Second, there is an increased inter-individual variability in several physiological parameters as well as markers of organ function (6–9). This makes the standardization of diagnostic and therapeutic algorithms, well entrenched in the *modus operandi* of current healthcare systems, hard to justify in this population. Moreover, it further limits a clinician’s ability to predict the short- and long-term outcomes of different patients suffering from similar disease conditions. Third, the same disease condition might present with fairly different symptoms and signs in an older patient vs. a young or a middle-age patient (10, 11). The clinical presentation is often characterized by vague and non-specific complaints, adding further complexity during the diagnostic process.

It is also worth noting that, with time, geriatricians have been actively managing an increasing number of disease conditions. The concept of “geriatric giants,” including falls, confusion, incontinence, and immobility, has been progressively replaced by one denoting a comprehensive assessment and management of patients often suffering from multiple chronic disease conditions. For example, patients with chronic obstructive pulmonary disease and/or heart failure, once managed primarily by respiratory physicians and cardiologists, attend geriatric services on a daily basis. Moreover, conditions previously unknown amongst older adults, e.g., acquired immunodeficiency syndrome, are becoming an increasing issue in this population. This has implications for disease management as well as detection and monitoring of drug-related toxicity (12, 13).

A comprehensive assessment and management plan for an older patient often requires concerted action by a number of additional professional groups such as nursing staff, physiotherapists, occupational therapists, rehabilitation physicians, and social carers. This multidisciplinary approach aims to provide longitudinal, proactive, and coordinated care to complex patients. However, limited access to these services might significantly prevent the accomplishment of such goals. In this regard, a recent UK report raises significant concerns about the social care funding crisis (14). The latter is the result of an increasing demand, particularly for people aged 85 years and over, as well as a reduction in public funding for social care. Funding projections, accounting for inflation, estimate that the same amount of care in 2010–2011 would cost GBP 2.57 billion more in 2015–2016 and GBP 4.45 billion more in 2020–2021. Perhaps more worryingly, such figures do not account for the inevitable increase in the older population, and the related demands, during the same period. Other developed countries are facing similar issues with reduced healthcare funding and staff shortages (15–18). Moreover, staff relocation to acute services in many healthcare organizations is likely to further affect the capacity to adequately plan, monitor, and ultimately deliver effective long-term care for older people suffering from complex disease conditions and various social circumstances.

Geriatric Medicine has played a key role in the development of dedicated clinical protocols and services, e.g., falls prevention clinics and programs, memory clinics, comprehensive geriatric assessment, and home visits, in different healthcare settings. Although there is good evidence of a meaningful clinical and socioeconomic impact for some of these services (19), some uncertainty remains with others (20, 21). This highlights the ongoing need for testing and monitoring established as well as novel management strategies in this complex population. An increased focus on older patients with multiple comorbidities as a more representative study group in clinical trials and clinical research has been advocated (22). However, there are a number of important issues that need to be considered, particularly in studies involving pharmacological agents (23). Trials in older people have focused on traditional, disease-specific, end-points, e.g., heart failure related hospitalizations or stroke recurrence. The clinical relevance of such outcomes is uncertain in a population characterized by significant inter-individual variability and complex patterns of co-morbidity (24). Instead, a number of “universal” outcomes such as dyspnea, fatigue, and physical function are likely to encompass a number of different, yet common, disease conditions, and to indicate a stronger impact of intervention strategies on overall wellbeing in this group. The use of unconventional end-points, however, carries potential issues with definition and standardization, particularly in the presence of cognitive impairment (25).

A key factor driving both current and future research agendas in healthy aging is the active involvement of end users, i.e., older adults, in identifying and refining research questions and outcomes of interest. According to two recent national surveys, conducted by the Disabled Living Foundation Charity in the UK and by the Home Instead Senior Care network in USA, respectively, older adults’ biggest fear is the loss of independence and moving into an institution, rather than dying (26, 27). Apart from the obvious consequences on psychological wellbeing and interpersonal relationships, loss of independence carries significant economic implications for healthcare systems. In USA, it has been estimated that the transition from being fully independent to needing help with ≥1 activity of daily living, but still living at home, increased the total annual health care spending per person from USD 4,771 to USD 18,025. The transition into a nursing home increased the annual cost to a staggering USD 36,596 (28).

Are there endogenous or external factors favoring the loss of independence, in addition to the consequences of well-known disease states, e.g., stroke, in older adults? Their identification might help developing better strategies to maintain an active and independent lifestyle in this population. Recent evidence suggests that iatrogenic factors, e.g., medications, frailty, and sarcopenia play a key role in this context. Prescribing of drugs with anticholinergic (antimuscarinic) and/or sedative effects has been shown to be independently associated with physical and cognitive decline. Moreover, it predicts adverse short- and long-term outcomes in older people (29–34). Changes in cognitive and physical function, often subtle, might be erroneously attributed to the aging process *per se*, rather than medications (35). Frailty and sarcopenia are also important markers of impaired homeostasis and susceptibility to either acute or chronic disease states (36, 37). Although there is no universal consensus on their definition, both frailty and sarcopenia independently predict adverse outcomes in older patient subgroups (38–41).

The emerging biological and clinical role of factors negatively affecting independence is likely to boost experimental, translational, and clinical research to identify the mechanisms involved as well as potential preventive and management strategies. Although “de-prescribing” interventions to reduce inappropriate exposure to anticholinergic and sedative drugs have been conducted with some success, their effects on cognitive and/or physical function are far from established (42, 43). Moreover, relatively little is known about the exact mechanisms responsible for the onset and progression of functional decline with these drugs. These important issues should be addressed in future studies. Similarly, current and novel experimental models of frailty and sarcopenia might lead to the identification of nutritional, pharmacological, and life-style interventions to be further tested in clinical trials (44, 45).

Geriatric Medicine and, more generally, research into aging face a number of challenges. The continuing expansion of the older population worldwide, together with the increasing exposure to new drugs and clinical conditions will require a regular review of the specialty aims, directions, work philosophy, and social impact. The further development, validation, and implementation of national curricula and core competencies will hopefully ensure a continuing interest of current and future generations of medical graduates in geriatric care and related medical specialties. This is particularly important at a time of economic uncertainty and resource allocation. Such challenges, however, might also represent exciting opportunities for identifying novel mechanisms of disease and frailty as well as testing pharmacological and/or non-pharmacological interventions on investigator – as well as patient-driven outcomes in adequate and representative study groups. As previously discussed, the identification of “universal” outcomes is likely to better reflect the impact of specific interventions on wellbeing and quality of life (24). Frontiers in Medicine, an innovative scientific medical journal and forum for ongoing debate amongst peers, is perhaps launched at the right time to facilitate some of these steps.

## Conflict of Interest Statement

The author declares that the research was conducted in the absence of any commercial or financial relationships that could be construed as a potential conflict of interest.
